# Successful treatment of *Listeria monocytogenes* prosthetic valve endocarditis using rifampicin and benzylpenicillin in combination with valve replacement

**DOI:** 10.1099/jmmcr.0.005085

**Published:** 2017-02-28

**Authors:** Tasnim Hasan, William Chik, Sharon Chen, Jen Kok

**Affiliations:** ^1^​ Centre for Infectious Diseases and Microbiology Laboratory Services, Institute of Clinical Pathology and Medical Research, Westmead Hospital, Westmead, New South Wales, Australia; ^2^​ Department of Cardiology, Westmead Hospital, Westmead, New South Wales, Australia; ^3^​ Centre for Research Excellence in Critical Infections, University of Sydney, Westmead Hospital, Westmead, New South Wales, Australia; ^4^​ Marie Bashir Institute for Infectious Diseases and Biosecurity, University of Sydney, Westmead Hospital, Westmead, New South Wales, Australia

**Keywords:** *Listeria*, endocarditis, prosthetic valve, rifampicin

## Abstract

**Introduction.** *Listeria monocytogenes* is an uncommon cause of prosthetic valve endocarditis (PVE). Recommended antimicrobial therapy typically includes intravenous β-lactams with or without synergistic aminoglycosides. *In vitro* studies have previously identified antagonism when rifampicin has been used in combination with β-lactams. However, *in vivo* data of rifampicin use are limited despite its enhanced anti-biofilm activity.

**Case presentation.** A 63-year-old male presented with fever and back pain. *L. monocytogenes* bacteraemia and bioprosthetic aortic valve endocarditis was confirmed, along with spinal discitis and osteomyelitis. He was successfully treated with benzylpenicillin and rifampicin, in conjunction with valve replacement.

**Conclusion.** Rifampicin remains an alternate agent to use, when there are contraindications to traditional aminoglycoside therapy. Further data on rifampicin use in *L. monocytogenes* PVE are awaited.

## Abbreviation

PVE, prosthetic valve endocarditis.

## Introduction

*Listeria monocytogenes* is a Gram-positive rod that can cause gastrointestinal and disseminated infection, in particular meningitis [1, 2]. Endocarditis is uncommon (only documented in approximately 80 cases), and has been reported in immunocompromised and competent hosts [1, 3]. Prosthetic valve endocarditis (PVE) and cardiac device infections caused by *L. monocytogenes* are rare [3].

In general, PVE has higher rates of morbidity and mortality compared to native valve endocarditis, particularly when associated with cardiac and cerebral complications [4]. Whilst there are recommendations for the management of PVE in general [4], specific guidelines on the management of PVE due to *L. monocytogenes* are limited. The role of surgery in *L. monocytogenes* PVE, including the timing and optimal method, is also unclear.

Historically, mortality from *L. monocytogenes* endocarditis was approximately 35 %, but more recently this has fallen to less than 15 % [1, 3]. Similar to the management of meningitis, recommended antimicrobial regimens typically include the use of intravenous β-lactams (ampicillin or benzylpenicillin), in combination with aminoglycosides (gentamicin or streptomycin).

In PVE cases caused by pathogens susceptible to rifampicin, this antimicrobial agent is commonly used given its enhanced anti-biofilm activity [5]. However, rifampicin is not commonly used in *L. monocytogenes* PVE as *in vivo* studies have shown antagonistic, rather than synergistic activity [6]. Herein, we report a case *of L. monocytogenes* PVE successfully treated with rifampicin, in combination with valve surgery. The case suggests that the addition of rifampicin does not cause harm and is not antagonistic, but can assist in the cure of PVE where dual anti-microbial therapy is often preferred for synergistic therapy.

## Case Report

A 63-year-old male presented to hospital with a 2 day history of fever and back pain. The patient had undergone a bioprosthetic aortic valve replacement 20 years earlier for rheumatic aortic valve disease, when he had concurrent coronary artery bypass surgery. His past history was also significant for hypertension, atrial flutter and type 2 diabetes mellitus.

Five days prior to the current admission, he had presented to hospital with diplopia, vertigo and syncope. A cerebral computed tomography scan revealed left frontal lobe and cerebellar infarcts; no intracranial haemorrhage was visualized. Blood cultures were not collected as he was afebrile. He was discharged after 48 h given the absence of significant neurological deficits. During the second presentation, *L. monocytogenes* was isolated from two sets of blood cultures collected on two consecutive days following 24 h incubation (penicillin MIC, 0.5 mg l^−1^; rifampicin MIC, 0.125 mg l^−1^). Magnetic resonance imaging of the spine revealed lumbar spine discitis and osteomyelitis at the L 4/L 5 level, associated with right paravertebral muscle enhancement but no epidural abscess.

**Fig. 1.**
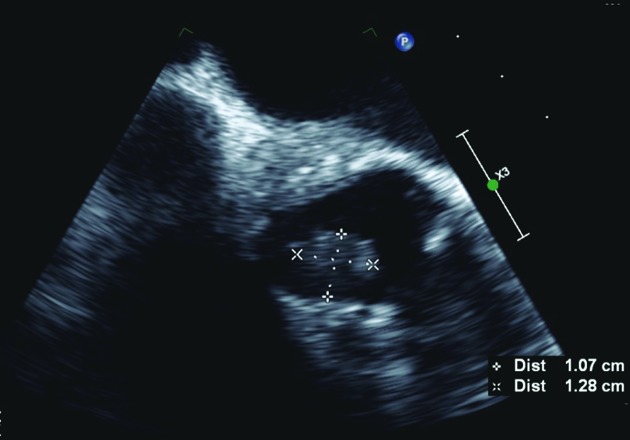
Transoesophageal echocardiograph of the bioprosthetic aortic valve.

Transoesophageal echocardiography showed a vegetation measuring 1.1×1.3 cm on the bioprosthetic aortic valve (Fig. 1) with possible aortic root abscess, with mild aortic regurgitation. He underwent aortic valve replacement on day 11 of admission. Histology of the resected valve showed necrosis with vegetation and pannus formation; no organisms were seen upon Gram staining or isolated from cultures.

The patient’s antimicrobial therapy included intravenous benzylpenicillin, 14.4 g per day for 6 weeks. Although the patient made a good recovery from his recent stroke, gentamicin was not used due to the potential for vestibular toxicity. Rifampicin, 300 mg twice daily, was prescribed instead. Following 4 weeks of therapy, rifampicin was stopped due to difficulties achieving a therapeutic level of anti-coagulation for the new mechanical aortic valve. After cessation of benzylpenicillin, the patient was commenced on amoxicillin, 1 g thrice daily, for treatment of his discitis and osteomyelitis for a total of 18 weeks. The C-reactive peptide level was initially 21 mg l^−1^ and the number of leucocytes was 10 000 cells µl^−1^. Inflammatory markers, however, normalized rapidly and remained normal throughout treatment.

No pathogens were isolated from multiple sets of blood cultures collected following the completion of antimicrobial therapy. At the 12 month follow-up, the patient had made a complete recovery.

## Discussion

Although rare, *L. monocytogenes* PVE can be treated successfully with antimicrobial therapy and surgery. However, *in vivo* data on the use of rifampicin in PVE caused by *L. monocytogenes* are limited, and its therapeutic role in this setting is unclear. We describe a case of *L. monocytogenes* PVE successfully treated with rifampicin used in combination with benzylpenicillin and surgery. Although timely valve replacement in the current case was fundamental to achieving a successful outcome, the early use of rifampicin prior to surgery was not detrimental and may have contributed to rapid recovery in the patient.

*Listeria* bacteraemia precedes the diagnosis of endocarditis in almost all cases [1, 3], and symptoms of cardiac failure or a history of rheumatic heart disease is common [1, 3]. In the present case, endocarditis was most definitely present during the patient’s first admission with embolic stroke. This case highlights the importance of collecting blood cultures even in the absence of fever, and giving consideration towards early echocardiography in patients presenting with stroke in the setting of prosthetic valves or cardiac devices.

In *L. monocytogenes* endocarditis, prosthetic valve involvement occurs in approximately one third of the cases [1]. Valve replacement was performed in approximately half of these cases, as in our case [1, 7]. In native valve endocarditis, surgery is often reserved for complicated cases, such as the presence of cardiac failure and large aortic root or para-valvular abscesses [2, 3].

For the treatment of *L. monocytogenes* endocarditis, the recommended duration of therapy ranges from 4 weeks in native valves to 6 to 8 weeks in prosthetic valves [1, 2, 7]. Our patient received 24 weeks of therapy in total for treatment of his discitis and osteomyelitis. Data are limited to guide therapy of *L. monocytogenes-*associated bone and joint infections, although in a case series of 43 patients a median of 15 weeks (range 2–88 weeks) of antimicrobial therapy was received by the patients; no treatment failures were observed in patients without a foreign device [8].

Antimicrobial therapy for *L. monocytogenes* infection typically includes β-lactams in combination with aminoglycosides [1, 2, 7], although definitive *in vivo* data for synergy is lacking. However, failure of therapy has been documented in PVE, when a single agent has been used [7]. This is concerning and evidence for alternatives to aminoglycosides is limited. Our case therefore provides a possible alternative in rifampicin therapy.

Rifampicin is an effective anti-biofilm active agent [5], particularly when used in combination with other agents [5]. This makes it an attractive agent for use in endovascular and prosthetic device infections, including PVE, where anti-biofilm active agents are important [5]. *In vitro*, *L. monocytogenes* is highly susceptible and bacteriostatic to rifampicin [9], and resistance is rare [9].

Despite this, *in vivo* data suggest that rifampicin is antagonistic when used in combination with β-lactams for the treatment of *Listeria* infections [6]. Although it is important to note that this may be within the initial few hours, as the killing rate of β-lactams with or without rifampicin is similar when measured at h [6]. However, synergy has been demonstrated when penicillin is used together with rifampicin on laboratory isolates [10].

To our knowledge, the use of rifampicin in *L. monocytogenes* endocarditis has only been reported once previously in the context of allergy to β-lactams. In that case, rifampicin was used in combination with teicoplanin and trimethoprim/sulfamethoxazole [11]. There is another case report of rifampicin use with ampicillin in an immunosuppressed patient with meningitis, where the isolate was resistant to trimethoprim/sulfamethoxazole, clindamycin and tetracycline [12]. At follow-up at 12 months and a few weeks, both patients in these cases were cured of their infections, as in the present case. These cases, together with our case, fail to demonstrate *in vivo* antagonism from rifampicin. In fact, our case progressed well with negligible inflammatory markers and stable clinical status throughout treatment.

The overall mortality for *L. monocytogenes* endocarditis is falling [1, 3], possibly due to increased recognition, earlier diagnosis and a multi-disciplinary team (including cardiologists, cardiothoracic surgeons and infectious diseases physicians) approach in the management of cases. Further data on the use of rifampicin for the treatment of *L. monocytogenes* PVE are awaited, and our case suggests that rifampicin may be an alternative to aminoglycosides for treatment, particularly where aminoglycoside use is contra-indicated.
